# Detection and Prevalence of Coronaviruses in European Bats: A Systematic Review

**DOI:** 10.1007/s10393-024-01688-5

**Published:** 2024-11-23

**Authors:** Mahima Hemnani, Priscilla Gomes da Silva, Gertrude Thompson, Patrícia Poeta, Hugo Rebelo, João R. Mesquita

**Affiliations:** 1 ICBAS—School of Medicine and Biomedical Sciences, Porto University, 4050-313 Porto, Portugal; 2 Epidemiology Research Unit (EPIunit), Institute of Public Health, University of Porto, Porto, Portugal; 3 Laboratório Para a Investigação Integrativa e Translacional em Saúde Populacional (ITR), Porto, Portugal; 4 LEPABE – Laboratory for Process Engineering, Environment, Biotechnology and Energy, Faculty of Engineering, University of Porto, Porto, Portugal; 5 ALiCE – Associate Laboratory in Chemical Engineering, Faculty of Engineering, University of Porto, Porto, Portugal; 6 CIBIO/InBIO, BIOPOLIS Program in Genomics, Biodiversity and Land Planning, CIBIO, Vairão, Portugal; 7 Microbiology and Antibiotic Resistance Team (MicroART), Department of Veterinary Sciences, University of Trás-os Montes e Alto Douro, 5000-801 Vila Real, Portugal; 8 Associated Laboratory for Green Chemistry (LAQV-REQUIMTE), University NOVA of Lisbon, 1099-085 Caparica, Portugal; 9 Veterinary and Animal Research Centre (CECAV), University of Trás-os Montes e Alto Douro, 5000-801 Vila Real, Portugal; 10 Veterinary and Animal Research Centre, Associate Laboratory for Animal and Veterinary Science (AL4AnimalS), Vila Real, Portugal

**Keywords:** Bats, *Alphacoronavirus*, *Betacoronavirus*, Europe

## Abstract

Bats are known hosts for a wide range of coronaviruses (CoVs), including those that cause severe acute respiratory syndrome (SARS-CoV-1) and Middle East respiratory syndrome (MERS-CoV). With the emergence of the COVID-19 pandemic caused by the SARS-CoV-2 virus, it has become increasingly important to understand the diversity and prevalence of CoVs in bat populations. This systematic review aimed to compile studies that have sampled CoVs from bats across Europe and assessed various aspects related to the testing of bat samples, including the country where the bats were collected, the CoV genomic region studied, the CoV genera that were detected, and the identification of bat species that were found to be carrying CoV. We identified 30 studies that assessed CoVs presence in bats across multiple countries including Italy, Germany, and various other nations with one or two studies each, which tested them for CoVs using a variety of matrices. CoVs were found in nine genera of bats, and the genomic regions included RdRp, ORF1a gene, as well as full genome, detecting α- and/or β-CoVs, with most of them being detectable only in faeces. This review provides a comprehensive overview of the CoVs detected in bats across Europe and highlights the importance of continued surveillance and monitoring of bat populations for potential emerging zoonotic CoVs.

## Introduction

Bats belong to the order Chiroptera, being one of the most diverse and abundant group of mammals, with over 1400 species identified (Bokelmann and Balkema-buschmann 2021; Wang et al. 2023). Bats are the only mammals that can fly and possess a diverse ecology allowing them to explore a vast array of habitats and roosts (Goffard et al. 2015), thus being found in different ecosystems all over the world, except for the Antarctica (Dacheux et al. 2014; Hu et al. 2017).

Bat species exhibit a diverse diet, including arthropods, insects, fruits, nectar, pollen, small mammals, fishes, frogs, and blood (Gloza-Rausch et al. 2008). They also found a niche to navigate and hunt in the darkness through the development of echolocation (Woo and Lau 2019; Nojiri et al. 2021). The ecological features of bats play crucial ecosystem services for the maintenance of environmental stability, acting as seed dispersers, pollinators, suppressors of insect populations, and nutrient recyclers (Kunz et al. 2011; Ramírez-Fráncel et al. 2022).

Evidence shows that bats are natural hosts of several disease-causing viruses across the globe (Wong et al. 2007; O’Shea et al. 2014), including zoonotic viruses such as Lyssavirus, Henipavirus, Marburgvirus, Ebolavirus, Astrovirus, Adenovirus, Filovirus, Paramyxovirus, and Coronavirus (Smith and Wang 2013; Moreno et al. 2017; Wang and Anderson 2019). Bats harbour specific traits that increase their chances of being virus reservoirs, such as an immune system that protects them against developing diseases, allowing them to maintain viruses in their bodies without evident clinical signs of infection (Bokelmann and Balkema-buschmann 2021; Ruiz-Aravena et al. 2022).

Besides this, ecological features can also facilitate the spread of viruses. Several bat species are highly social forming some of the largest colonies of vertebrates, thus creating the conditions for a facilitated spread of viruses within bat populations (Kerth 2008). Bats often share roosts such as tree cavities with other animals and/or settle close to humans, and this may affect the chance of spreading viruses between host species due to the closer physical contact in dense roosts (Falcón et al. 2011; Steyer et al. 2013; Kemenesi et al. 2015; Betke 2023), therefore increasing the chances of a zoonotic spillover. The main threats faced by bats are habitat loss, damage to roosts, and hunting (IUCN Species Survival Commission 2001; Red Latinoamericana y del Caribe para la Conservación de los Murciélagos RELCOM 2010).

Coronaviruses (CoVs) are viruses belonging to the order Nidovirales, *Coronaviridae* family, subfamily *Orthocoronavirinae.* They are a positive-sense single stranded RNA virus, with one of the largest RNA genomes (Bokelmann and Balkema-buschmann 2021), and usually have an envelope equipped with protruding protein structures on their surface called spikes (Domańska-Blicharz et al. 2021). CoVs have diverse animal hosts ranging from mammalian to avian species, causing mainly enteric and respiratory diseases of varying severity (Balboni et al. 2011). CoVs are classified into four genera. *Alphacoronavirus* (α-CoV) and *Betacoronavirus* (β-CoV), which typically cause diseases in mammals, are considered pathogenic viruses. On the other hand, *Gammacoronavirus* (γ-CoV) and *Deltacoronavirus* (δ-CoV) evolved from a coronavirus originated from birds, and the majority of them (but not exclusively) cause diseases in birds (Mihindukulasuriya et al. 2008; Woo et al. 2012, 2014), and are known as poultry viruses (Gloza-Rausch et al. 2008).

Since CoVs are found in a wide variety of hosts, including wildlife, livestock, poultry, pets, and humans, these viruses are considered to have a high potential for interspecies transmission (Reusken et al. 2010). Moreover, due to their long genomes of 30 kb, high recombination frequency, and high mutation rate, CoVs have the potential to adapt to new host species with novel pathogenicity and cause a wide spectrum of diseases (Woo et al. 2009). The most iconic examples of viral spillover to humans have occurred in 2002/2003, when a highly pathogenic CoV (SARS-CoV-1) emerged in China, causing worldwide outbreaks (Peiris et al. 2004). A few years later, in 2012, an even more deadly CoV emerged in the Arabian Peninsula (MERS-CoV), with a case-fatality rate of 34.3% (Zumla et al. 2015). By the end of 2019, a new SARS-related CoV named severe acute respiratory syndrome CoV-2 (SARS-CoV-2) emerged, leading to the COVID-19 pandemic. To date, the world has paid a high toll in this pandemic in terms of human lives lost, economic losses, and increased poverty (Ciotti et al. 2020).

Several studies indicate that bats might be the natural and primary reservoirs for several viruses closely related to other mammalian CoVs and that mammalian CoVs were derived from ancestor viruses found in bats (Woo et al. 2009; Platto et al. 2021; Ruiz-Aravena et al. 2022). They are also considered the group of mammals harbouring the largest number of CoVs (Bokelmann and Balkema-buschmann 2021), and the evolutionary source for several human coronaviruses (Ruiz-Aravena et al. 2022). Alpha and beta CoVs have been detected in bats from 14 to 21 bat families in at least 69 countries across six continents (Ruiz-Aravena et al. 2022). The absence of CoVs in particular bat taxa is most likely due to insufficient sampling rather than true absence (Anthony et al. 2017; Wang et al. 2023).

To predict risks for host transition and disease outbreak, therefore, it is important to understand the nature of CoVs hosts, their association with certain bat species, and the association between bat species (Gloza-Rausch et al. 2008). It is also important to understand colony size, density, and composition, as it could affect virus prevalence by changing transmission rates both within and between roosts. Reproductive cycles also influence prevalence and transmission of viruses in bat colonies by affecting patterns of behaviour and physiological susceptibility (Serra-Cobo and López-Roig 2017; Chaverri et al. 2018; Ruiz-Aravena et al. 2022). Research into the origins of SARS-CoV-2 and continuing interest in CoVs ecology and evolution have highlighted the value of bat surveillance (Cohen et al. 2023).

Any microorganism has the potential to become airborne in certain environmental situations (i.e. to be present in aerosolized particles), posing serious threats to the health and well-being of both human and animal populations (Verreault et al. 2008). Phylogenetic analysis has shown that SARS-CoV-2 has 79–79.5% genetic similarity with SARS-CoV-1, making it a SARS-related CoV (Contini et al. 2020; Lu et al. 2020; Rothan and Byrareddy 2020; Zhou et al. 2020). Considering that prior research on these viruses has indicated the possibility of airborne transmission (Olsen et al. 2003; Zhao et al. 2011; Pyankov et al. 2018; Tellier et al. 2019; Ramanathan et al. 2020; da Silva et al. 2021; Spencer et al. 2024), other SARS-related CoVs with the capability to spread via the airborne route (Geng and Zhou 2021) could be potentially harboured by bats.

Across Europe, there are approximately 55 species of bats (Russo 2023) belonging to six families: *Emballonuridae*, *Molossidae*, *Pteropodidae*, *Rhinolophidae, Vespertilionidae*, and *Miniopteridae*. In this continent, the vast majority of bats are insectivorous consuming beetles, flies, moths, and other insects, although there are a couple of exceptions where *Rousettus aegyptiacus* feeds on fruit and *Nyctalus lasiopterus* also preys on small birds (Russo 2023). In Europe, certain bat species are of particular interest with respect to CoVs. They play a crucial role in pest regulation, while some also function as pollinators and aid in the dispersal of seeds for numerous plants that hold significance for humans (Afelt et al. 2018; Lu et al. 2021). Some bats, such as the common pipistrelle and the greater horseshoe bat, and others display different ecological roles and behaviours that have ramifications for CoV ecology and evolution (Gonzalez and Banerjee 2022). These differences in diet, ranging from insects to small mammals, and roosting behaviour, which can include solitary roosts or groups of hundreds to thousands of individuals, create unique environments conducive to CoV maintenance and transmission (Gannon and Bovard 2016; Gonzalez and Banerjee 2022). Some of these species, such as those that move across the continent such as the *Pipistrellus nathusii*, could move viruses over long distances (Shipley et al. 2019; Alcalde et al. 2021). Others species, like those that form dense colonies, such as the greater mouse-eared bat (*Myotis myotis*), might increase virus transmission events among and between species (Calisher et al. 2006; Lazov et al. 2018; Ruiz-Aravena et al. 2022). Despite significant advancements in understanding bat-associated CoVs, there are some research gaps regarding specific European bat species and their role in CoV transmission (Luis et al. 2013; Phelps et al. 2019; Olival et al. 2020). Geographic gaps also exist, with certain regions in Europe lacking comprehensive surveys for bat-associated CoVs, potentially overlooking important reservoirs.

The primary challenges faced by European bats encompass the loss, degradation, and fragmentation of their habitats, overuse of pesticides, as well as societal intolerance, lack of awareness, developmental activities, and persecution (Ruiz-Aravena et al. 2022). These factors result in the decline of bat colonies found in various locations such as buildings, trees, underground sites, and bridges, along with the depletion of areas abundant in insects for their feeding, disturbances, and direct persecution of bat populations. Understanding the interactions of these bats with humans and livestock, especially in regions where they frequently share habitats, is crucial for assessing zoonotic spillover risks.

In recent years, both α-CoVs and β-CoVs have been detected in bats of different species in several European countries. Vespertilionidae and Rhinolophidae are the most likely ancestral host families for α-CoVs in Europe, and β-CoVs likely originated in Vespertilionidae (Russo 2023)*.* To our knowledge, until now only one systematic review has been conducted on bat CoVs (Cohen et al. 2023), which primarily highlighted the importance of sample type, repeat sampling, and longitudinal study design as key predictors of coronavirus prevalence. However, this study did not fully address the need for high-resolution data stratification by specific factors such as location, bat species, virus species or strain, and sample type. To provide baseline data to inform future surveillance efforts, our systematic review aims to fill these gaps by providing a detailed analysis of the presence and characterization of CoVs in European bats. Understanding these dynamics is crucial for informing public health policies and strategies.

## Methodology

The present review includes studies published from1st January 2008 until 22nd May 2024, in the following databases: PubMed, Web of Science, and Scopus. The Preferred Reporting Items for Systematic Reviews and Meta-Analysis (PRISMA) criteria were followed for the systematic review (Shamseer et al. 2015), and the studies considered must be published, indexed, and peer reviewed. Language barriers were included, only articles written in English were considered for this review. The terms used for searching were “bat”, “coronavirus”, and designating each European country.

We evaluated which bat matrices were analysed, in which country the bats were sampled, which CoV genomic region was studied, which CoV genera were found in the bats, and which bat species were identified shedding CoV. After reading the title and the abstract, papers that did not address the detection of CoVs in bats in any European country in the scope or part of the scope were excluded from this systematic review. Unclear information in the title and abstract was a factor leading to the read in full, and only those that contained the target content were included. Two independent investigators (MH and PGdS) screened the databases, and relevant information was extracted. Differences in opinions about whether to include an article were solved by consensus between the authors.

## Results and Discussion

**Figure 1:**
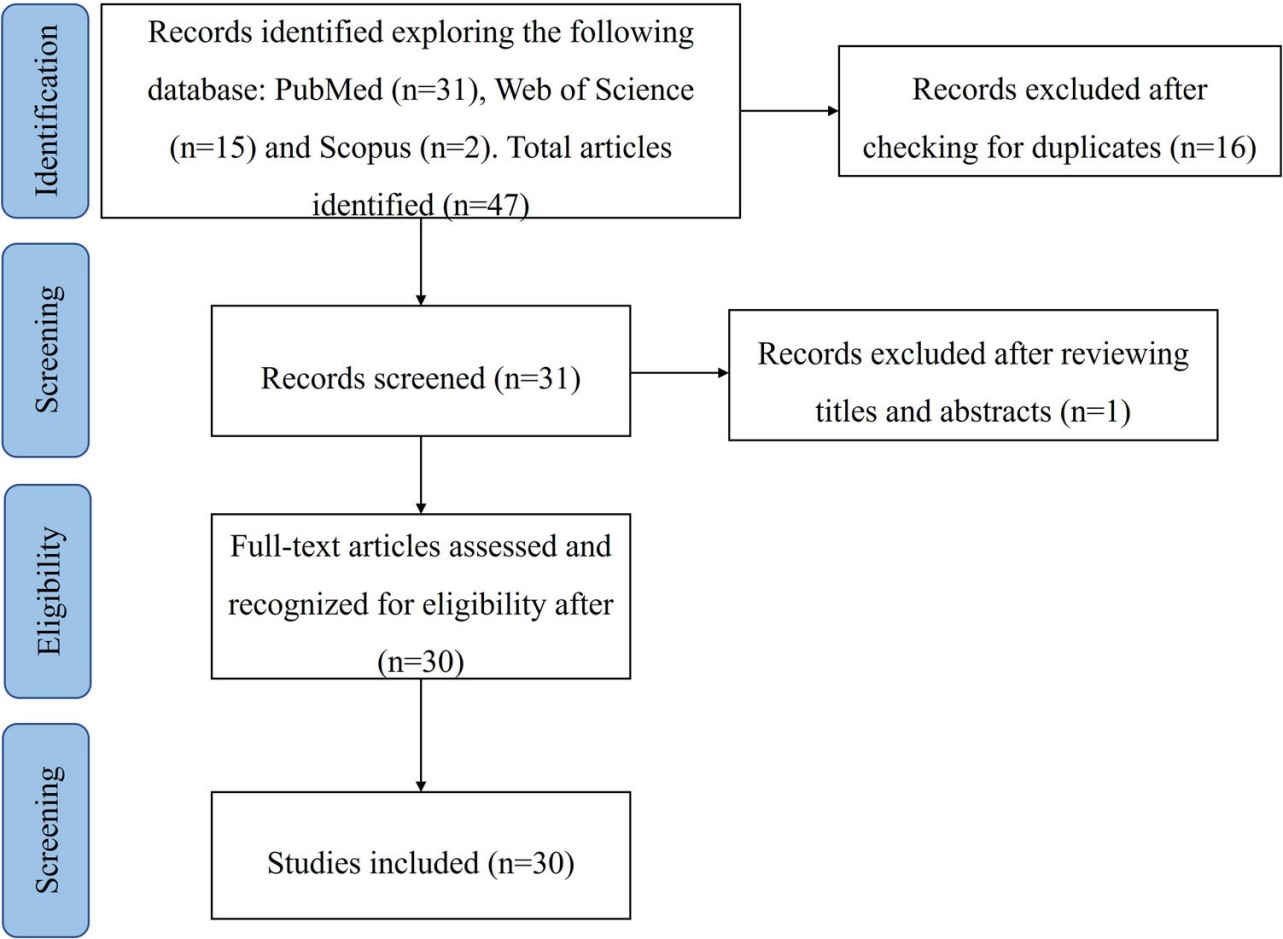
Preferred Reporting Items for Systematic Reviews and Meta-Analyses (PRISMA) flowchart.

A total of 33 articles with potential interest were found in the databases used for the search (Fig. 1) and screened for the extraction of relevant information. Application of inclusion and exclusion criteria allowed the identification of 32 papers potentially suitable for the systematic review, and one was excluded based on the criteria that its aim was not finding which CoVs were harboured by the bats. After a complete read of the papers, 30 of them were considered eligible and included in this review.

The chosen publications assessed various aspects related to the testing of bat samples, including the country where the bats were collected, the CoV genomic region studied, the CoV genera that were detected, and the identification of bat species that were found to be carrying CoV, and the main information is compiled in Table 1.

**Table 1:** Compilation of studies that sampled CoVs in bats across Europe, including the country of each study, matrices tested, genomic region studies, which CoV genera were found and the bat species that were identified with CoV.

Reference	Country of study	Matrices	Genomic region analysed	CoV genera	Bat species identified with CoV
(Gloza-Rausch et al. 2008)	Germany	Faeces	RdRp	Group 1	*Myotis bechsteinii; M. dasycneme; M. daubentonii; Pipistrellus nathusii; P. pygmaeus* (Vespertilionidae)
Rihtarič et al., 2010	Slovenia	Faeces	RdRp	β-CoV	*Rhinolophus hipposideros* (Rhinolophidae)
(Reusken et al. 2010)	Netherlands	Faeces	RdRp	α-CoV	*M. daubentonii; M. dasycneme. P. pipistrellus; Nyctalus noctule* (Vespertilionidae)
(Drexler et al. 2010)	Bulgaria	Faeces	RdRp		*R. ferrumequinum; R. hipposideros; R. euryale; R.s mehelyi; R. blasii;* (Rhinolophidae) *Miniopterus schreibersii; N. leisleri* (Vespertilionidae)
(Balboni et al. 2011)	Italy	Faeces and/or Anal swabs	RdRp	β-CoV	*R. ferrumequinum* (Rhinolophidae)
(Drexler et al. 2011)	Germany	Faeces	RdRp	α-CoV	*Myotis myotis* (Vespertilionidae)
(Falcón et al. 2011)	Spain	Faeces, oral swabs	RdRp	α-CoV and β-CoV	*Eptesicus isabellinus; Hypsugo savii; Miniopterus schreibersii; M. blythii; M. daubentonii; M. myotis; N. lasiopterus; P. kuhlii; P. sp* (Vespertilionidae)
(August et al. 2012)	UK	Faeces	RdRp	α-CoV	*M. natteri; M. daubentonii* (Vespertilionidae)
(Lelli et al. 2013)	Italy	Faeces, brain, intestine, viscera	RdRp	α-CoV and β-CoV	*R. hipposideros* (Rhinolophidae)*; N. noctula; H. savii P. kuhlii* (Vespertilionidae)
(Annan et al. 2013)	Germany, Netherlands, Romania, and Ukraine	Faeces	RdRp	β-CoV	*P. nathusii; P. pipistrellus; P. pygmaeus* (Vespertilionidae)
(Van Gucht et al. 2014)	Belgium	Lung/ Intestine	RdRp	None	*None*
(Kemenesi et al. 2014)	Hungary	Faeces	RdRp	α-CoV and β-CoV	*M. daubentonii; M. Myotis; M. natteri; P. pygmaeus* (Vespertilionidae)*; R. euryale; R. ferrumequinum; R. hipposideros* (Rhinolophidae)
(De Benedictis et al. 2014)	Italy	Anal swabs	RdRp	α-CoV and β-CoV	*M. blythii; E. serotinus* (Vespertilionidae)
(Goffard et al. 2015)	France	Guano	RdRp	α-CoV	*P. pipistrellus*(Vespertilionidae)
(Fischer et al. 2016)	Germany	Saliva, faeces, urine	RdRp		*M. nattereri; P. nathusii; P. pygmaeus; M. bechsteinii*(Vespertilionidae)
(Monchatre-Leroy et al. 2017)	France	Intestine	RdRp		*P. pipistrellus; P. sp; M. emarginatus; M. nattereri; Miniopterus schreibersii* (Vespertilionidae)
(Yurchenko et al. 2017)	Ukraine	kidney, heart, lungs	Full genome	None	*None*
(Pauly et al. 2017)	Luxembourg	Faeces	RdRp	α-CoV and β-CoV (SARS-related)	*R. ferrumequinum* (Rhinolophidae)*; M.* *emarginatus* (Vespertilionidae)
(Moreno et al. 2017)	Italy	Pools of viscera (lungs, heart, spleen, liver, and intestine)	Full genome	β-CoV (MERS-related)	*P. kuhlii; H. savii* (Vespertilionidae)
(Lazov et al. 2018)	Denmark	Faeces	ORF1b	α-CoV	*M. daubentonii; M. dasycneme; M. nattereri; P. pygmaeus; E. Serotinus* (Vespertilionidae)
(Lecis et al. 2019)	Italy (Sardinia)	Faeces/ oral swabs	RdRp	β-CoV	*R. ferrumequinum* (Rhinolophidae)*; Plecotus auritus; Tadarida teniotis* (Vespertilionidae)
(De Sabato et al. 2019)	Italy	Faeces and carcasses	RdRp	α-CoV	*P. kuhlii* (Vespertilionidae)
(Kivistö et al. 2020)	Finland	Faeces and guano samples	RdRp	α-CoV and β-CoV	*E. nilssonii; M. brandtii; M. daubentonii* (Vespertilionidae)
(Lwande et al. 2022)	Sweden	Blood, saliva, and faeces	RdRp	α-CoV	*M. daubentonii* (Vespertilionidae)
(Cholleti et al. 2022)	Sweden	Saliva and faeces	Full genome	α-CoV	*Pipistrellus pygmaeus* (Vespertilionidae)
(Wiederkehr et al. 2022)	Switzerland	Faeces	Full genome	β-CoV (MERS-related)	*Rhinolophus hipposideros* (Rhinolophidae)*, Myotis myotis, Vespertilio murinus* (Vespertilionidae)
(Speranskaya et al. 2023)	Russia	Faeces	Full genome	β-CoV (MERS-related)	*P. nathusii* (Vespertilionidae)
(Tan et al. 2023)	UK	Faeces	Full genome	α-CoV and β-CoV (MERS-related)	*M. daubentonii, P. pipistrellus, P. pygmaeus, P. auritus* (Vespertilionidae)*, R. ferrumequinum, and R. hipposideros* (Rhinolophidae)
(Hemnani et al. 2023)	Portugal	Air, faeces, anal/oral swabs	RdRp	α-CoV	*Miniopterus schreibersii, Myotis myotis* (Vespertilionidae)*, Rhinolophus mehelyi, Rhinolophus ferrumequinum* (Rhinolophidae)
(Hemnani et al. 2024)	Portugal	Faeces	RdRp	α-CoV	*Pipistrellus pipistrellus* (Vespertilionidae)

RdRp—RNA-dependent RNA polymerase; α-CoV—*alphacoronavirus*; β-CoV—*betacoronavirus*; ORF—Open Reading Frame.

**Figure 2:**
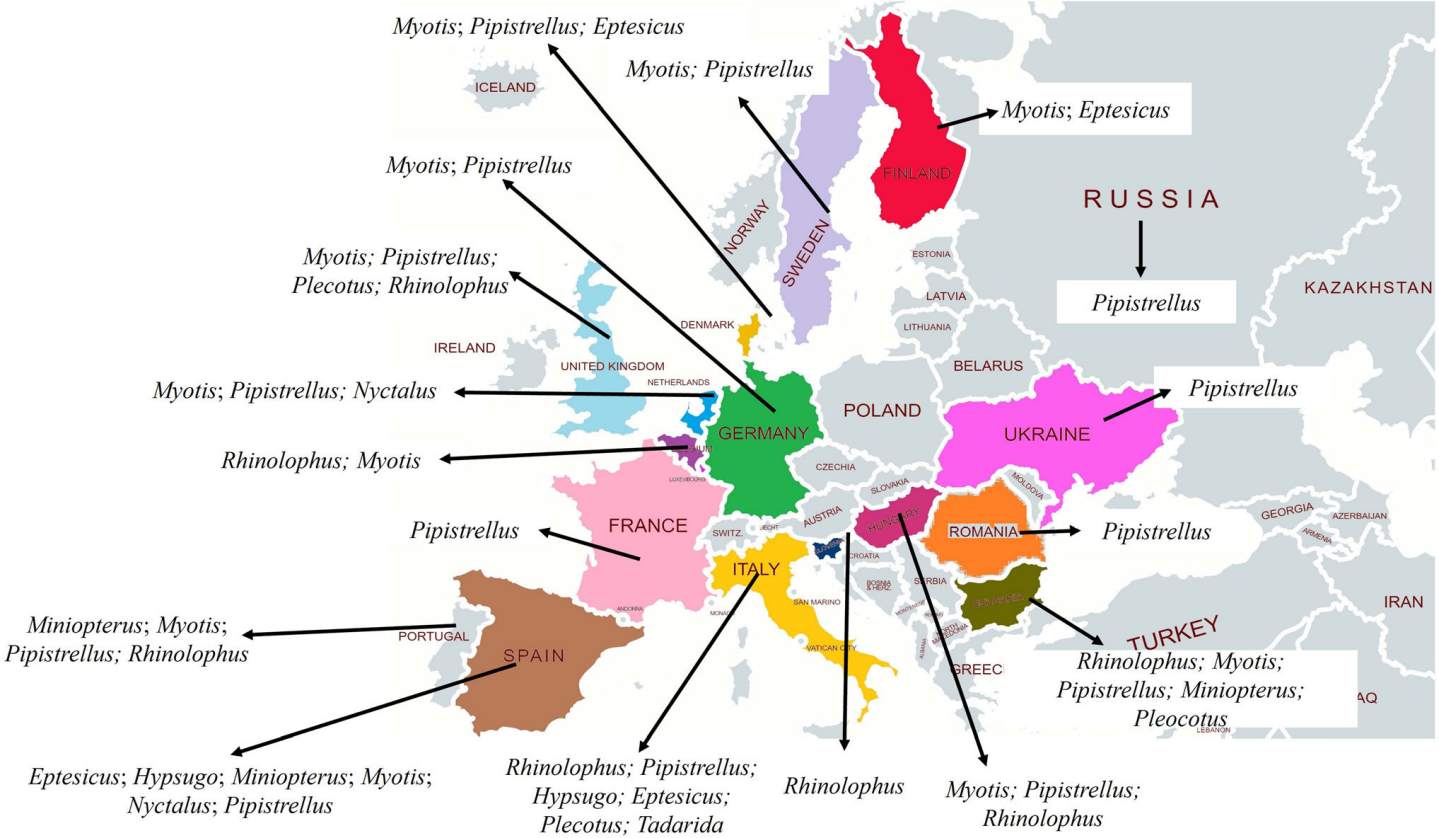
Bats genera that were found carrying CoVs in European countries.

From the screening of articles, 30 studies evaluated the presence of CoVs in bats in Europe, with six articles from Italy, four from Germany, two from the Netherlands, two from Ukraine, two from the UK, two from Sweden, two from Portugal, and one from Belgium, Bulgaria, Denmark, Finland, France, Hungary, Luxembourg, Romania, Slovenia, Spain, Switzerland, and Russia each. Bat genera in which CoVs were found according to each country are compiled in Figure 2.

In total, CoVs were found in nine genera of bats belonging to the families Rhinolophidae, Vespertilionidae, and Miniopteridae: *Rhinolophus, Myotis*, *Pipistrellus*, *Plecotus*, *Tadarida*, *Nyctalus*, *Hypsugo*, *Eptesicus*, and *Miniopterus*, totalling 26 species (see Table 1) from the approximately 55 species that can be found in Europe. The studies collected oral and anal swabs, faeces, guano, saliva, blood, brain, kidney, heart, lungs, intestine, pools of viscera (lungs, heart, spleen, liver, and intestine), and carcasses. Most studies (n = 23) assessed the presence of CoVs targeting the RdRp gene, while six assessed the full genome and one assessing the ORF1b gene. The CoVs detected were α- and β-CoVs, and most of them were only detectable in faeces. One study assessed the presence of CoVs in the air from bat environments, but the results were negative.

*Myotis daubentonii*’s strains found in the UK were related more than 70% to sequences from the same bats from Germany (August et al. 2012); *Nyctalus noctula* strains found in Italy clustered with the strains from another bat from the same genera found in Spain (*Nyctalus lasiopterus*), but also clustered with another bat (*Eptesicus serotinus*) found in Italy; the strains from the bats found in France clustered with the ones from Germany, Luxembourg, and UK (Supplementary Fig. 3). Sequence analysis conducted on the acquired CoV sequences from Portugal in caves, and tree-dwelling habitats revealed significant similarities to sequences obtained from bats discovered in Bulgaria, Italy, Spain, and UK (Hemnani et al. 2023, 2024). The identities ranged from 93 to 100%, indicating a close relationship between the CoV strains circulating in European bats. The exception was the strains found in bats from Germany (Gloza-Rausch et al. 2008) that were closely related with a clade of viruses from Chinese bats, and the strains found in bats from Bulgaria were found to closely related to CoVs previously found in bat species from China and Hong Kong (Drexler et al. 2010). The CoVs identified in bats from Luxembourg (*Myotis emarginatus* and *Rhinolophus ferrumequinum*) had a high degree of similarity to SARS-CoV (> 98% nucleotide identity) (Pauly et al. 2017), while the CoVs from bats from Italy (*Pipistrellus kuhlii* and *Hypsugo savii*) had > 80% similarity to MERS-CoV. Research from the UK on the *Plecotus auritus* bat (Tan et al. 2023) indicates a relationship to MERS-CoV-like merbecoviruses isolated from *Hypsugo*, *Pipistrellus*, and *Vespertilio* spp. in Western Europe and China. Additionally, a study from Russia shows that the *Pipistrellus nathusii* bat falls into a distinct subclade closely related to human and camel MERS-CoV (Speranskaya et al. 2023). The genetic links between bat CoVs and MERS-CoV variants suggest ongoing vigilance is crucial to prevent potential outbreaks and protect public health against emerging infectious diseases.

**Figure 3:**
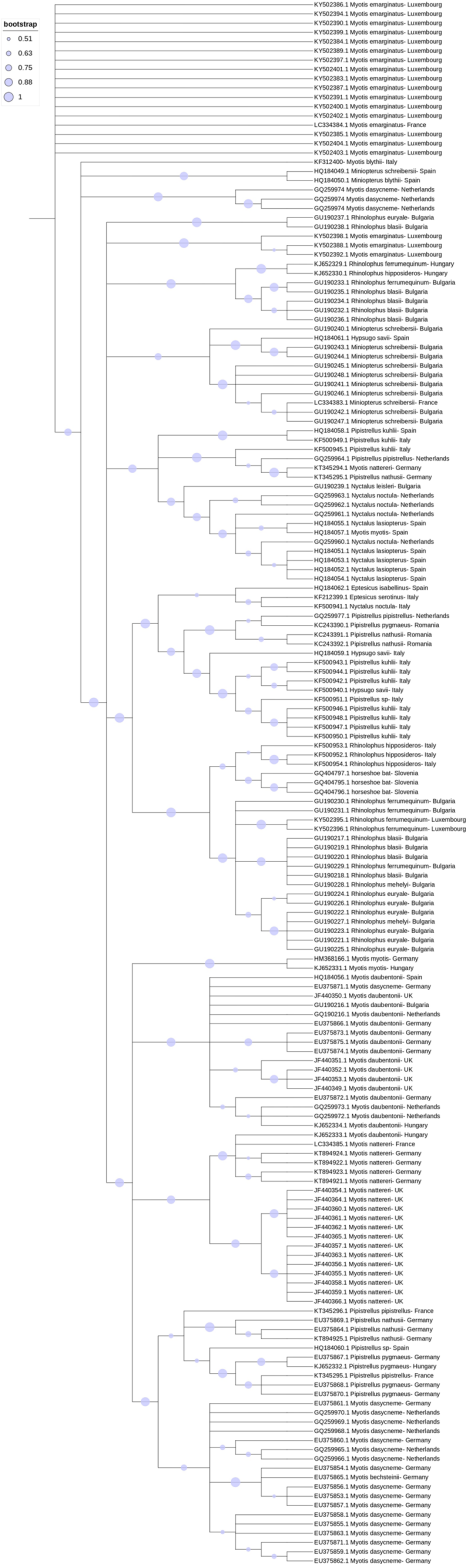
Phylogenetic tree constructed for the α- and β-CoVs strains identified in the 24 studies approached in this revision. Phylogenetic analysis was based on a 406 nt partial region of the RdRp. The tree was constructed using MEGA 10 using the maximum likelihood based on the GTR + G model, and 1000 bootstraps were replicated.

**Figure 4:**
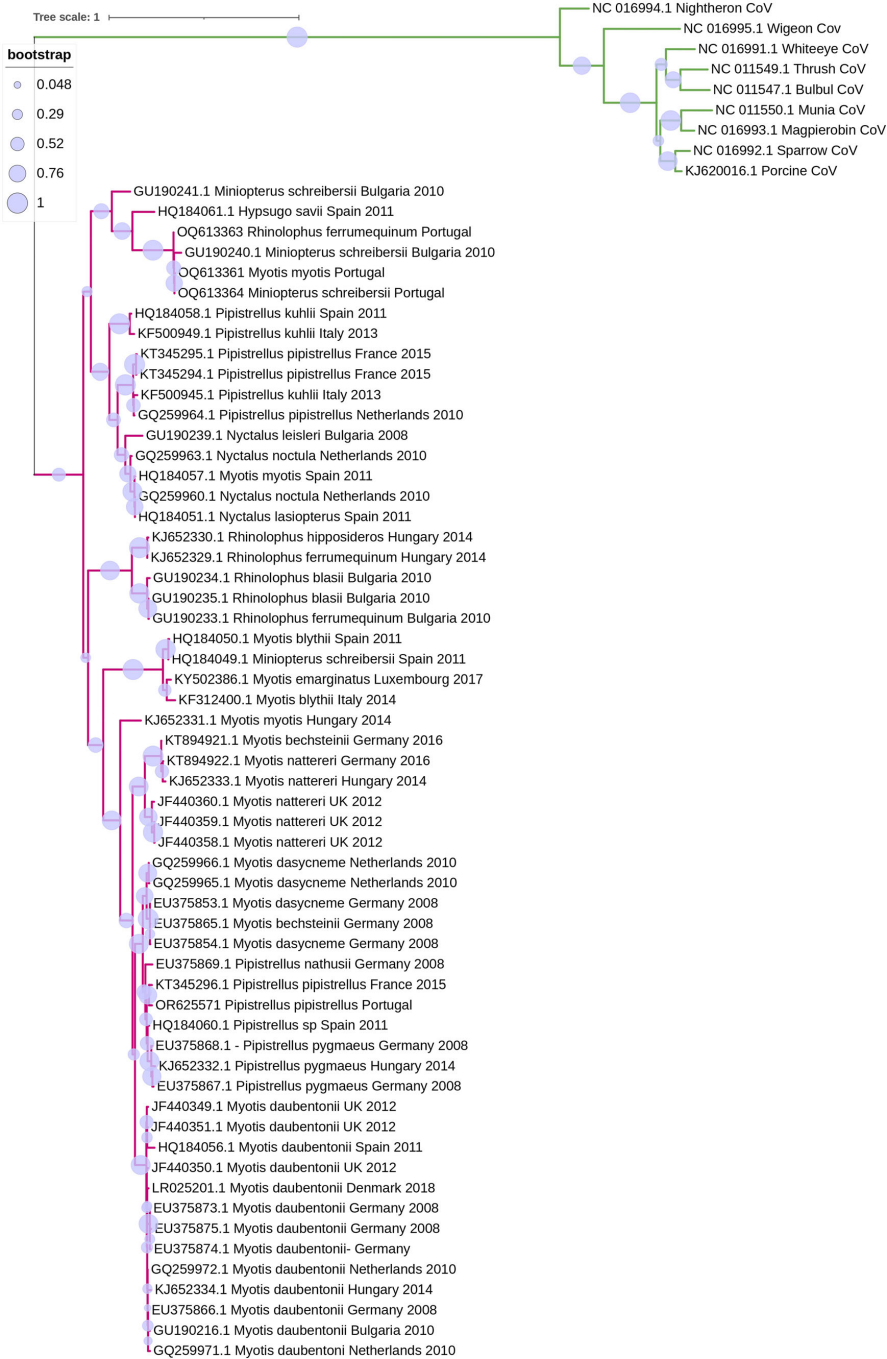
Phylogenetic tree constructed for the α-CoVs strains identified in this revision. Phylogenetic analysis was based on a 406 nt partial region of the RdRp. The tree was constructed using MEGA 10 using the maximum likelihood based on the GTR + G model, and 1000 bootstraps were replicated. Samples from this study are indicated in pink with the description of GenBank accession number, host bat species, the county where it was found, and the year. In green are the γ-CoV, an outgroup for the analysis.

**Figure 5:**
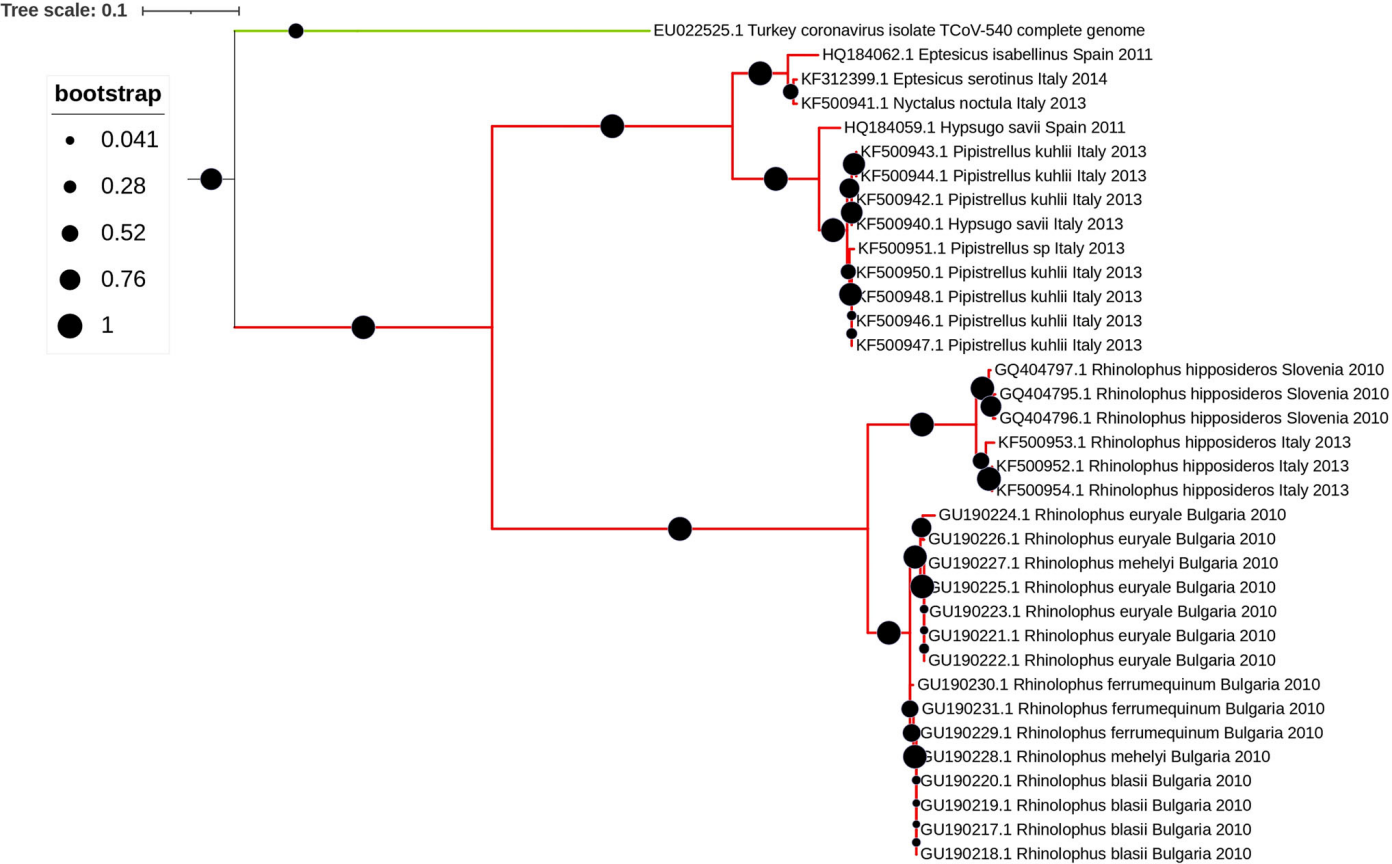
Phylogenetic tree constructed for the β-CoVs strains identified in this revision. Phylogenetic analysis was based on a 406 nt partial region of the RdRp. The tree was constructed using MEGA 10 using the maximum likelihood based on the GTR + G + I model, and 1000 bootstraps were replicated. Samples from this study are indicated in pink with the description of GenBank accession number, host bat species, the county where it was found, and the year. In green is a Turkey-CoV strain, a γ-CoV, an outgroup for the analysis.

Interestingly, published studies reported that CoV strains within each geographic location clustered together, which could suggest a potential co-evolution between CoVs and the bat host (Ruiz-Aravena et al. 2022). On the other hand, the co-hibernation of different bat species in one location has been reported to facilitate the spillover of viruses between genera/species (Calisher et al. 2006; Lazov et al. 2018). Studies have also reported that CoV strains from European bats clustered with strains found in Chinese bats, another suggestion would be that the viruses’ strains are not exclusively correlated to the geographic location, but can also be associated with the bat species, as they use similar niches across the world (Reusken et al. 2010). The convergence of CoV strains from European and Chinese bats, despite geographic separation, underscores the potential role of shared ecological niches, such as forests and caves, in facilitating viral transmission among bat populations worldwide. Figures 3 and 4 show a phylogenetic tree of the RdRp region constructed for the alpha and beta CoV strains, respectively, found in the papers assessed in this revision. As it can be noticed in Figure 4, *Myotis daubentonii*’s CoV strains clustered with α-CoVs strains from different geographic locations like Hungary, Bulgaria, UK, Germany, but it can also be noticed in Figure 5, strains from different species from the same genera *Rhinolophu*s clustered in the same geographic location. This suggests that similar CoV strains detected can be found in the same bat genera rather than clustered within each geographic location, but there is also clustering of strains within the same geographic location originated from bats from different species, suggesting there is no rule or pattern of transmission.

Bats are also known to have regular migration, although migration information is much lower than for other terrestrial vertebrates such as birds due to many reasons such as the size of these mammals and their nocturnal activity (Vasenkov et al. 2023). It is well-documented that some bat species can carry out long migrations. For example, the *Nyctalus noctula* bat that can be found in the remote north-eastern part of Europe can fly through central European countries to southern Europe (Vasenkov et al. 2023). Additionally, there are also bats like *Nyctalus* spp. that often fly over several hundred kilometres, though rarely more than 1000 (Popa-Lisseanu and Voigt 2009). These migrations can consequently facilitate a co-infection between bats and spillover between bats and other animals. While European bat migrations are relatively well-understood (Hutterer et al. 2005), there is a need for further research on bat migration patterns in other geographic locations to fully comprehend the global dynamics of bat-borne diseases.

When it comes to the presence of bat CoVs in the environment, all the studies published until this date only tested faeces, blood, urine, and saliva. Only one study performed air sampling in a bat habitat (bat caves in this case) in order to assess whether there is presence of CoVs or other viruses in the air of the sampled environments (Hemnani et al. 2023). Although the study did not have any positive air sample for bat CoVs, a few things should be considered when interpreting these results. First, negative results do not necessarily mean that there was no virus present at the time of collection, as viral concentrations in the air could have been low at the location and time of collection. Therefore, a longer sampling period might be necessary to collect enough airborne viruses for modern molecular tools to be able to detect them (Lednicky et al. 2020). Secondly, environmental factors like temperature, relative humidity, and wind currents should also be taken into account as they may influence the outcomes (Tang 2009; Pica and Bouvier 2012; Spencer et al. 2024). Third, there are different air sampler devices available for air sampling. These include filter-based, impingers, impactors, cyclones, and water-based condensation samplers (Verreault et al. 2008). In this study, a Coriolis® Compact air sampler that concentrates airborne particles using a dry cyclonic technique was used (Carvalho et al. 2008). Additionally, the effectiveness of different air sampling devices remains uncertain due to a lack of comparative studies and standardized protocols.

To predict risks for CoVs host transition and disease outbreaks, we need to have a deeper understanding of the nature of CoVs hosts, including the association of certain CoVs with bat species (Gloza-Rausch et al. 2008). Studying bats in various geographic locations and different species of bats can assist in identifying and recognizing the range of hosts and the potential role of these CoVs in causing new infectious diseases. These findings provide insight into bat CoV evolution and ecological transmission among bat taxa. The identified hotspots of bat CoV evolution and transmission will guide early warnings of bat-borne CoV zoonotic diseases (Wang et al. 2023).

As shown earlier, bats form colonies with many individuals and can fly long distances, increasing the spread of viruses in many geographic locations to other bats and to humans and other livestock or wilds animals, without or with very little influence in the bat immune system (Bokelmann and Balkema-buschmann 2021). Bats have been identified as a significant source of various emerging viruses, including CoVs. Due to the vast genetic variation and the wide range of locations of CoVs discovered in bats, there is a high probability of future disease outbreaks (Wang and Anderson 2019).

In numerous parts of the world, bats are labelled with a negative reputation, and due to the COVID-19 pandemic, there is an excessive amount of fear towards them. Misunderstandings of the relationship between COVID-19 and bats might be additionally fuelled by the long-credited role of bats in causing human diseases (O’Shea et al. 2014; Fenton et al. 2020). Bats are frequently associated with zoonotic diseases and have been suggested to carry more viruses than other mammalian groups (Drexler et al. 2012; Lu et al. 2021). This negative perception and fear are adversely affecting their populations, leading to harmful actions against them and neglect of their ecological importance. As a result of the negative perception and fear towards bats, they are increasingly vulnerable to a range of human threats. These threats include well-documented habitat loss (Lu et al. 2021) which increases human contact with bat populations and leads to direct bat deaths (Platto et al. 2021). Moreover, the misunderstanding of the bat–virus relationship can result in harmful actions against bats, such as culling, and can inadvertently enhance the spread of viruses to humans and other domesticated animals (Rupprecht et al. 2023). Therefore, it is crucial to correct public misunderstandings about the bat–virus relationship to prevent direct harm to bat survival and to recognize their ecological importance (Lu et al. 2021).

In conclusion, this systematic review highlights the significant role of bats as reservoirs for a diverse array of CoVs, including those related to significant past outbreaks such as SARS and MERS. SARS-CoV-2, the virus behind the COVID-19 pandemic, was identified in late 2019 in Wuhan, China, and is believed to have originated from bats, similar to other CoVs like SARS, MERS, and several human CoVs. Some CoVs, however, may have emerged from rodents, and notable outbreaks such as swine acute diarrhoea syndrome (PEDV) in pigs also trace back to bat origins. While our review focuses on bat populations, further comparative studies are needed to understand the prevalence of these pathogens in bats relative to other potential host reservoirs. This understanding is crucial for comprehensively assessing the zoonotic risk and devising effective mitigation strategies. Against the backdrop of the ongoing global COVID-19 pandemic caused by the SARS-CoV-2 virus, our synthesis of 30 studies in multiple European countries illuminates the dynamics of CoV prevalence in bat populations. The identified genomic regions, including RdRp, ORF1a gene, and full genome, coupled with the detection of α- and/or β-CoVs in nine genera of bats, accentuates the need for a nuanced understanding of the zoonotic potential residing within these species. The extensive distribution and variety of CoVs in bats can be attributed to genetic mutations and recombination, as observed in bat migration patterns (suggesting a long co-evolutionary relationship), along with the bats' ability to host infections and co-infections. The recurrent identification of CoVs in bat faces serves as a reminder of the intricate ecological interactions driving viral transmission.

Despite these findings, significant gaps remain. Future studies could focus on longitudinal studies to monitor CoV prevalence over time, investigate the impact of habitat changes on viral dynamics, and explore the interactions between different bat species and other wildlife (Cohen et al. 2023). Such research could explain more how CoV prevalence changes over time, identify any seasonal patterns or trends, and determine the factors that influence these changes. It could also help assess the impact of human activities and how human interventions, such as habitat destruction or conservation efforts, affect the prevalence and transmission of CoVs. It is also important to understand colony size, density, composition, and reproductive cycles, as these factors could affect virus prevalence by changing transmission rates both within and between roosts, and the prevalence and transmission of viruses in bat colonies by affecting patterns of behaviour and physiological susceptibility (Ruiz-Aravena et al. 2022).

Viruses can be found in different surfaces and biological fluids, as well as in the air (Labadie et al. 2020). While a variety of factors, including the virus's ability to replicate in a naive host and contact with that host, are often involved in viral transmission (Geoghegan et al. 2016), the survival of a virus shed into the environment is also a necessary condition for its spread (Bosch et al. 2006; Geoghegan et al. 2016; Kumar et al. 2021). In order to better understand the dynamics and behaviour of the aerosolized virus, a longer sampling period might be necessary to collect enough airborne viruses for modern molecular tools to be able to detect them (Lednicky et al. 2020). It is also important to take into account environmental factors that may impact the outcomes such as temperature, relative humidity, and wind currents (if any). Also, test the effectiveness of different air sampling devices for different environments. Future studies should aim to address these variables to enhance our understanding of virus aerosolization in bat environments.

Future studies should also focus on viral metagenomics, as it is a target-free and unbiased approach that has proved to be a powerful tool for characterizing viral nucleic acids from any type of sample and for identifying unknown viruses (Paez-Espino et al. 2016). Additionally, this method can also be employed to explore the bat virome from various sample matrices, bat species, and locations, integrating longitudinal studies with metagenomics.

The establishment of a continuous veterinary surveillance programme for detecting CoVs in bat populations across numerous countries may also help us comprehend the ecology of new CoV infections (Lelli et al. 2013) contributing to global efforts aimed at mitigating the impact of emerging infectious diseases.
